# Spontaneous Gastric Intramural Hematoma: Case Report and Literature Review

**DOI:** 10.7759/cureus.23268

**Published:** 2022-03-17

**Authors:** Omar Tabbikha, Hani Maalouf, Christina Abou-Malhab, Ribal Aby Hadeer, Raja Wakim

**Affiliations:** 1 General Surgery, Mount Lebanon Hospital Balamand University Medical Center, Beirut, LBN

**Keywords:** case report, gastric hematoma, gastric intramural hematoma, gastric submucosal tumor, spontaneous gastric intramural hematoma

## Abstract

Spontaneous gastric intramural hematoma is a rare disease. Herein, we present a case of a previously healthy 28-year-old male patient who presented with diarrhea and diffuse abdominal pain of one-week duration. The patient was diagnosed with spontaneous gastric intramural hematoma post urgent partial gastrectomy for a bleeding gastric tumor. Six other cases of spontaneous gastric intramural hematoma are published in the literature; therefore, when encountering a case of intra-abdominal mass attached to the gastric wall, gastric intramural hematoma should be considered in the differential even when no cause is present.

## Introduction

Gastric intramural hematoma (GIH) is a very rare non-cancerous lesion. Most gastrointestinal intramural hematoma described in the literature are found in the esophagus or duodenum [1,2]. While the most frequent cause of GIH is found to be coagulopathies, other etiologies that have also been reported in the literature include complications of endoscopy, vascular aneurysms, peptic ulcer disease, amyloidosis, fishbone ingestion, pancreatitis, spontaneous, and others [3].

The rarity of this disease especially with the absence of a trigger can make the diagnosis of spontaneous gastric intramural hematoma (SGIH) very challenging. To our knowledge, only six cases of SGIH are reported in the literature, of which four were managed surgically and two conservatively [4-9]. Herein we present a rare case of a previously healthy 28-year-old male patient who underwent laparotomy for a bleeding gastric mass that turned out to be an SGIH.

## Case presentation

A 28-year-old male patient, previously healthy, presented to our institution for severe abdominal pain of one-day duration. The history goes back to one week prior to presentation when he started to develop watery diarrhea associated with mild diffuse crampy abdominal pain, nausea, one episode of non-bilious vomiting, one episode of subjective fever, chills, night sweats, decrease in appetite, unintentional weight loss of 9 kgs in five months, but no melena or hematochezia. The patient self-diagnosed himself with gastroenteritis and took rifaximin for three days followed by metronidazole for another three days but symptoms didn’t resolve. 

One day prior to presentation, the patient developed severe abdominal pain that was diffuse, cramping in nature, constant, 10/10 in intensity, radiating to his back and shoulders, exacerbated by movement, and with no relieving factors. He also developed several episodes of presyncope described as lightheadedness, blackout, and diaphoresis with a documented systolic blood pressure reaching as low as 85mmHg so he presented to the emergency room (ER) in a peripheral hospital for evaluation. To note, the patient takes no medications or illicit drugs, has no allergies, is a social smoker, drinks alcohol occasionally, has a negative family medical history, and has previously undergone a right inguinal lymph node dissection about three years previously that turned out to be reactive in nature with no signs of malignancy. 

**Figure 1:**
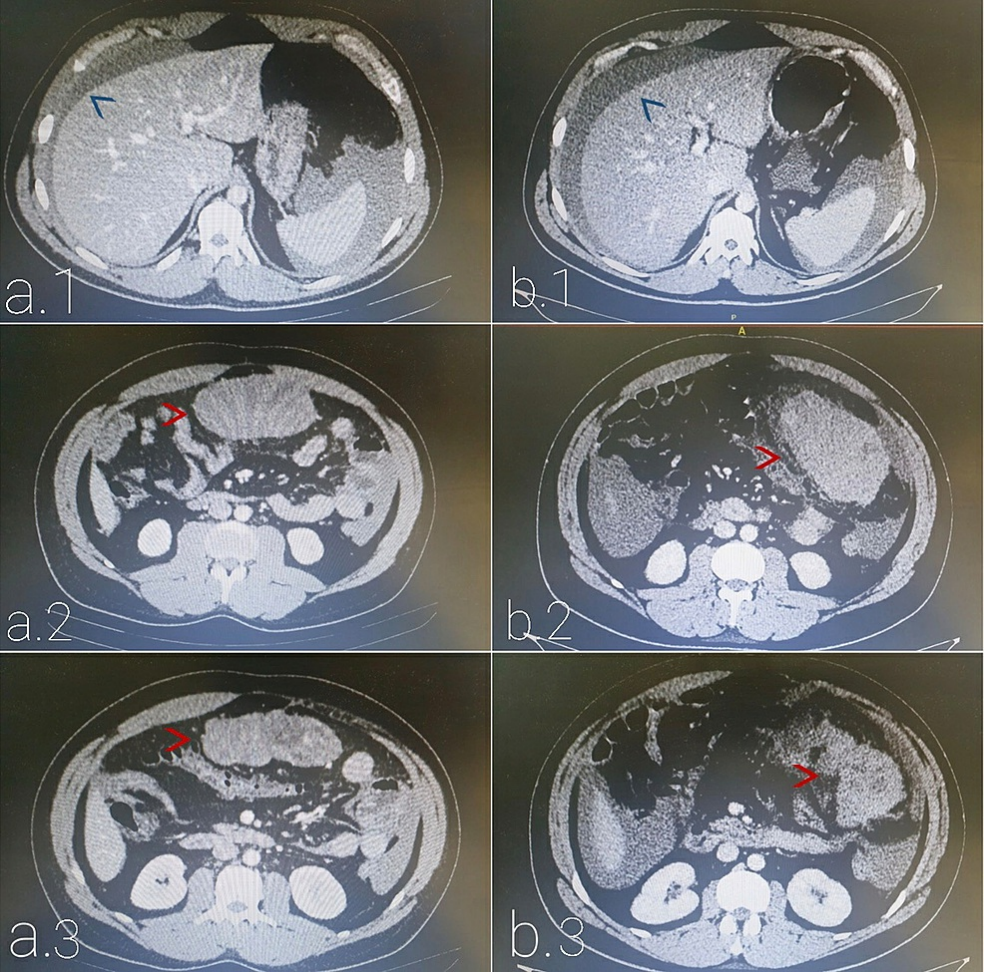
CT Scans of Abdomen and Pelvis a (1-3): CT scans with IV contrast done in the peripheral hospital. b (1-3): Urgent CT scans with IV contrast done at our institution. Blue arrow: perihepatic fluid; red arrow: gastric lesion

In the peripheral hospital, the patient was hydrated and laboratory tests were unremarkable except for a white blood count of 18.6 x 10^3/ul with neutrophils of 88%. Computed tomography (CT) scan of the abdomen and pelvis with IV contrast (Figure 1a) demonstrated an anterior abdominal mass of 10.4x8.1x4.2 cm dimensions. The mass is located along the greater omentum, possibly from the outer gastric wall, along the midline. It has heterogeneous density with mild heterogeneous enhancement, few prominent vascularity, well-defined lobulated borders, and no calcification or fat components. The mass is inseparable from the gastric greater curvature serosa with clear fat planes separating it from adjacent small bowels and transverse colon. Few small adjacent lymph nodes measuring less than 1 cm were noted with mild ascites located predominantly in the pelvis, peri-splenic, peri-hepatic, and lower paracolic gutters slightly dense suggesting complicated ascites or hemorrhage. The patient presented to our institution for further investigation.

**Figure 2:**
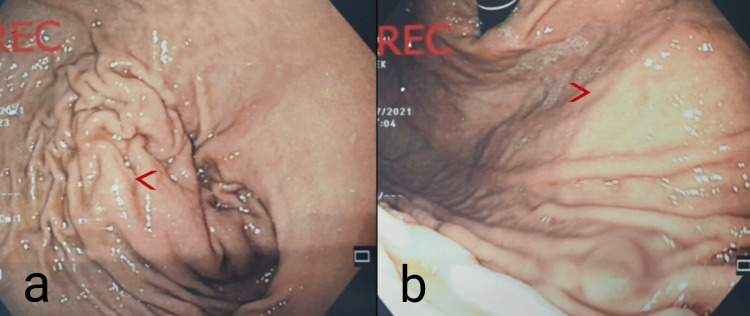
Gastroscopy Showing Highly Suspected Submucosal Lesion Gastroscopy displaying a highly suspected submcosal lesion (a); the second panel shows the same from another angle (b). Red arrow: area with suspected submucosal mass

In our ER, his vitals were stable. During the physical exam, he was pale, had abdominal guarding, severe diffuse abdominal tenderness, and mild distension. Laboratory tests showed no pertinent changes in values except for a noted hemoglobin drop from 14 g/dl to 11.2g/ dl. The patient was hydrated, started on IV antibiotics, and was admitted to the facility. Gastroscopy (Figures 2a-2b) showed a highly suspected submucosal lesion with an irregular shape measuring about 6 cm between the body and antrum with normal esophagus and duodenum. The biopsy taken showed negative findings. Endoscopic ultrasound (EUS) with biopsy and ascitic TAP with cytology were planned but before being fulfilled the patient’s abdominal pain increased tremendously in intensity, became tachycardiac (heart rate reaching 126 bp) and his hemoglobin further dropped, reaching 7.3g/dl. 

An urgent CT scan of the abdomen and pelvis with IV contrast was done (Figure 1b) that showed hyper-dense areas in the previously noted abdominal mass suggesting a bleeding tumor with irregular contours and surrounding fat stranding suggestive of peri-tumoral fissuration / micro-perforation with an increase in previously noted ascites but no pneumoperitoneum. At this point, our working diagnosis was a ruptured gastrointestinal stromal tumor (GIST). The patient was transfused with two units of packed red blood cells and a decision for urgent laparotomy was made. 

**Figure 3:**
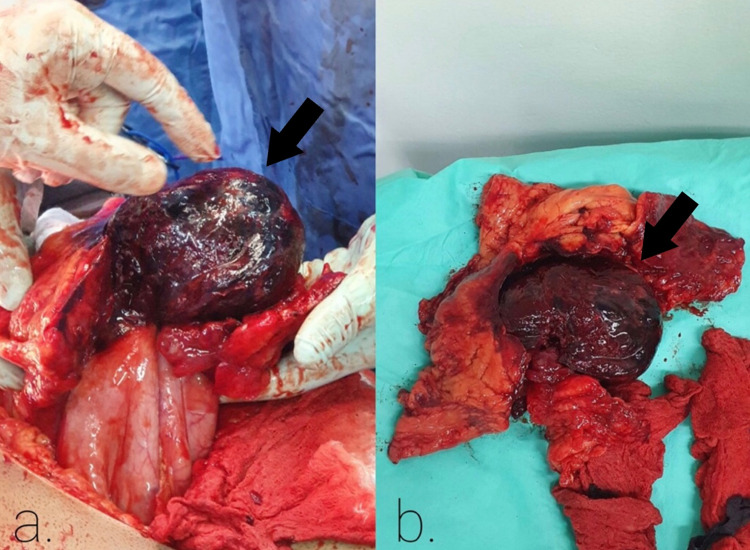
Surgical Specimen (a) Gastric mass (black arrow) identified in the body and antrum of the gastric greater curvature (b) The partial gastrectomy surgical specimen containing the identified lesion (black arrow)

On laparotomy, two liters of blood were aspirated from the abdomen and a bleeding gangrenous gastric mass was identified in the body and antrum of the greater curvature (Figure 3a) in contact with the mesocolon of the transverse colon. A wedged resection of the denoted gastric lesion (Figure 3b) with appropriate margins (partial gastrectomy) was made, followed by excision of the mesocolon directly in contact with the mass and all of these specimens were sent to pathology. Post-operatively, the patient's stay was not complicated and the final pathology turned out to be GIH.

## Discussion

The stomach is an uncommon location for gastrointestinal intraluminal hematoma but when present it is usually in patients who take anticoagulants, have gastric pathologies, experienced endoscopy, or rare cases, spontaneously [3]. The rarity of GIH may lead in many cases to its exclusion from the differential diagnosis especially when it’s spontaneous and in a previously healthy patient (as in our case), since there will be no focus for it to be brought to the physician’s attention; consequently, this may result in misdiagnosis, mismanagement, or the execution of aggressive procedures in a benign pathology. Therefore, to document, evaluate, and stress the possibility of SGIH in patients with no triggering factors we conducted an extensive PubMed search looking for all the published case reports in the English literature of patients found to have SGIH; our findings are summarized in Table 1. 

**Table 1:** The Reported Cases of Spontaneous Gastric Intramural Hematoma in The Literature M: male, F: female, N/A: not applicable

First Author	Age/ Sex	Chief complaint/ Presentation	Primary diagnosis/ Differential diagnosis	Surgical vs Conservative Management	Reason for surgery	Type of surgery	Diagnosis of gastric intramural hematoma
Hui et al [4].	49/ M	Severe epigastric pain	hemorrhagic gastric lesion/ perforated peptic ulcer, acute pancreatitis	Surgical	Ongoing bleed/ gastric mass	Total gastrectomy	Postoperative
Costa et al. [5]	75/ F	Melena	partially thrombosed aneurysm of the splenic artery, pancreatic cystic neoplasm with gastric invasion, pancreatic pseudocyst complicated with hemorrhage.	Surgical	Gastric mass	Radical subtotal billroth 2 gastrectomy	Postoperative
Rajagopal et al. [6]	N/A	N/A	N/A	Conservative	N/A	N/A	N/A
Oe et al. [7]	74/ M	Nausea and hematemesis	Gastroesophageal submucosal hematoma, gastric varices, tumor or extrinsic mass compression/ malignant melanoma	Conservative	N/A	N/A	Post resolution
Yoshioka et al. [8]	60/ F	Left flank pain	Rupture of visceral artery aneurysm	Surgical	Ongoing bleeding	Gastric wedged resection with 1 cm margin	Postoperative
Spychała et al. [9]	26/ M	Lower abdomen pain	Gastrointestinal stromal tumor	Surgical	Gastric mass	Total gastrectomy with roux-en-y anastomosis	Postoperative
Current case	28/ M	Diffuse abdominal pain	Gastric submucosal tumor/ gastrointestinal stromal tumor, gastric lymphoma	Surgical	Ongoing bleeding	Partial gastrectomy with abutting mesocolon resection	Postoperative

Even though GIH is mostly asymptomatic, complaints such as nausea, blood-stained vomit, or epigastric pain can be present; moreover, hematomas can impact the osmotic gradient within the gastrointestinal tract wall which can lead to hemorrhagic effusion into the peritoneal cavity [2]. The chief complaints in the published case reports of SGIH are various but the most common presentation noted is abdominal pain (as in our case) even though its location is nonspecific. For instance, the published cases include patients presenting with abdominal pain in the epigastrium [4], lower abdomen [9], and the left flank [8] while in our case the pain was diffuse. Other chief complaints included melena, hematemesis, and nausea [5,7].

CT scan is considered the most specific and sensitive in diagnosing intramural hematomas [1]. The hematoma will appear as a well-defined, hyper-dense mass that does not infiltrate the adjoining structures and has no calcifications which is a characteristic finding in cancer lesions [10]. However, we noticed that CT scan may still lead to misdiagnosis especially with SGIH since CT scan when used didn’t find the right diagnosis in any of the published cases. For instance, a CT scan in our case was made for evaluation; however, the findings were suggestive of a gastric submucosal mass - mainly GIST - which was also the case in two of the reported cases [4,9] while in a third case the diagnosis was ruptured visceral aneurysm [8] and only postoperatively the pathology showed GIH. 

CT angiography can also be used in the diagnosis of GIH since it can detect active hemorrhage or the presence of a visceral arterial aneurysm in the vicinity of the stomach. In case of an active hemorrhage, angiography can also facilitate the initiation of treatment by embolization of the damaged vessels [10]. In the cases that turned out to have SGIH, two had visceral aneurysms as the working diagnosis, and CT angiography did not lead to the correct diagnosis [5,8]. In our case, CT angiography was not considered since our patient presented to our institution with CT scans suggesting a non-bleeding gastric submucosal mass. On commencement of bleeding, the clinical scenario necessitated urgent laparotomy.

Gastroscopy can also play a role in the investigation of GIH. It can show a gastric luminal narrowing with or without submucosal bright-red or dark-red mass and rarely with the presence of active mucosal bleeding [10]. In the reported cases, endoscopy was done only in three of them [5,7,9] but did not suspect the right diagnosis except in one case [7]. For instance, in this case, gastroscopy showed a very large dark red mass in the cardia with sharply rising edges; therefore, a gastroesophageal submucosal hematoma was included in the differential but the diagnosis was not certain until endoscopic ultrasound with biopsy was done - ruling out malignancy - and until the lesion was resolved conservatively with follow up. In our case, a gastroscopy was done and it showed no lesions, ulcerations, mucosal bleeding, or irritation but we highly suspected a submucosal lesion.

EUS can help in the diagnosis of GIH. Indeed, it can facilitate the determination of the wall thickness from which the lesion originates and assess its echostructure and the thickness of its infiltration. Moreover, it permits the physician to perform thin-needle or core-needle biopsy to collect the material for cytological and histopathological analyses [11]. EUS with biopsy was used in only one of the mentioned cases and the biopsy result yielded hemorrhagic tissue with no malignancy [7]. This finding allowed the physician to follow conservative management and the diagnosis of SGIH which was only confirmed after the resolution of the lesion [7]. In our case, we were first suspecting submucosal lesion so, a EUS with biopsy was ordered but the patient’s conditions deteriorated before its fulfillment.

There is still no agreed modality for the treatment of SGIH; however, GIHs are considered in general self-restrained and the majority of them are reabsorbed within two to three weeks [1,3]. In GIH, the managing clinician should first aim to resuscitate the patient and secure hemostasis [3]. Conservative treatment is used mainly in patients with coagulopathies and their treatment consists of administration of clotting factors and blood transfusions [1,11]. In case of failure of conservative treatment, reported therapeutic modalities to achieve hemostasis include arterial embolization, endoscopic and percutaneous drainage, and surgery [3,8]. 

In case of active bleeding or a trend toward enlargement, transcatheter arterial embolization (TAE) may be indicated [10]; however, it is only technically possible if contrast extravasation of the defected artery is identified. For instance, in the cases of SGIH, TAE was initiated in only one case when the working diagnosis was rupture of a visceral aneurysm; however, angiography of only the superior and inferior mesenteric artery was done (not the celiac artery) showing no extravasation of the contrast agent and consequently moving on with surgical resection [8]. 

In general, surgical management of GIH is recommended where there is unclear diagnosis, suspected complications, hematoma involving a large part of the gastric wall, ongoing bleeding, failure of minimally invasive therapy, or when one cannot differentiate it from malignant gastric tumors; moreover, the decision to operate emergently and kind of surgery is mainly driven by the clinical scenario [9,12]. In four of the six reported cases of SGIH surgical management was done, and in all of them, the diagnosis of intramural hematoma was post-operation. In two of the surgically managed cases, the reason for surgery was ongoing bleeding and the cause of bleeding was ascribed to be a gastric mass [4,8]. In the other two, the reason was to resect an identified gastric mass [5,9]. In one of the cases where surgery was done for ongoing bleeding, total gastrectomy was done due to the identification of a mass in the fundus and body with gangrenous patchy changes in the lesser curvature [4]; however, in the second case, the working diagnosis was an impending rupture of visceral artery aneurysm but intraoperatively a gastric submucosal mass was suspected to be located in the antrum, so wedged resection of the mass was done with 1 cm margins [8]. In the cases where surgery was driven due to the presence of gastric mass, radical subtotal Billroth 2 gastrectomy was done in one of them [5], while in the second case a total gastrectomy with roux-en-Y anastomosis was done since the mass was covering the entire posterior wall of the stomach extending from the antrum to the pylorus [9]. In our case, an urgent CT showed a bleeding gastric mass; therefore, an urgent laparotomy was decidedly the next step. Intraoperatively, a bleeding gangrenous gastric mass was identified in the body and antrum of the greater curvature in contact with the mesocolon; hence, partial gastrectomy was done with resection of the mesocolon abutting the gastric mass and also, as in all the other cases, the diagnosis of SGIH was done postoperatively. 

It is noted that in the majority of the cases of SGIH and in all the cases that were treated surgically, the working diagnosis, whether preoperatively or intraoperatively, was gastric submucosal mass. Nevertheless, because of the dilemma in the diagnosis, these patients may have received unnecessary gastrectomies or aggressive surgical procedures (as in mesocolon resection in our case) for a benign pathology that was identified postoperatively in all the cases. 

In the reported cases, the differential diagnosis was broad including (other than gastric submucosal tumor) visceral artery aneurysm, compression from extrinsic mass, gastric varices, pancreatic pseudocyst, and others. For instance, only one case suspected GIH and it was treated conservatively [7]. Since our patient was previously healthy and based on his CT scans, age, and the physicians’ assessment, a gastric submucosal tumor (mainly GIST) was the working diagnosis. Gastric lymphoma was included in our differential due to the presence of B-symptoms but was considered less likely based on the imaging studies. GIH was not included in our differential due to its rarity and due to the absence of a focus for it to be brought to our attention. The pathologic study of the surgical specimen showed no focus for the hematoma and the etiology of the hematoma is considered spontaneous.

## Conclusions

SGIH is a very rare disease. When suspected in a patient presenting with an intra-abdominal mass attached to the gastric wall, GIH should be taken into consideration and included in the differential. As our review showed, this is true even in young and healthy patients with no cause for the disease. Diagnosing SGIH is of great importance, since it is a benign etiology that can be treated conservatively avoiding unnecessary aggressive surgical procedures.
